# Describing the antimicrobial usage patterns of companion animal veterinary practices; free text analysis of more than 4.4 million consultation records

**DOI:** 10.1371/journal.pone.0230049

**Published:** 2020-03-13

**Authors:** Brian A. Hur, Laura Y. Hardefeldt, Karin M. Verspoor, Timothy Baldwin, James R. Gilkerson

**Affiliations:** 1 Asia-Pacific Centre for Animal Health, Melbourne Veterinary School, University of Melbourne, Parkville, Victoria, Australia; 2 School of Computing and Information Systems, University of Melbourne, Parkville, Victoria, Australia; 3 Centre for the Digital Transformation of Health, University of Melbourne, Parkville, Victoria, Australia; University of Lincoln, UNITED KINGDOM

## Abstract

Antimicrobial Resistance is a global crisis that veterinarians contribute to through their use of antimicrobials in animals. Antimicrobial stewardship has been shown to be an effective means to reduce antimicrobial resistance in hospital environments. Effective monitoring of antimicrobial usage patterns is an essential part of antimicrobial stewardship and is critical in reducing the development of antimicrobial resistance. The aim of this study is to describe how frequently antimicrobials were used in veterinary consultations and identify the most frequently used antimicrobials. Using VetCompass Australia, Natural Language Processing techniques, and the Australian Strategic Technical Advisory Group’s (ASTAG) Rating system to classify the importance of antimicrobials, descriptive analysis was performed on the antimicrobials prescribed in consultations from 137 companion animal veterinary clinics in Australia between 2013 and 2017 (inclusive). Of the 4,400,519 consultations downloaded there were 595,089 consultations where antimicrobials were prescribed to dogs or cats. Antimicrobials were dispensed in 145 of every 1000 canine consultations; and 38 per 1000 consultations involved high importance rated antimicrobials. Similarly with cats, 108 per 1000 consultations had antimicrobials dispensed, and in 47 per 1000 consultations an antimicrobial of high importance rating was administered. The most common antimicrobials given to cats and dogs were cefovecin and amoxycillin clavulanate, respectively. The most common topical antimicrobial and high-rated topical antimicrobial given to dogs and cats was polymyxin B. This study provides a descriptive analysis of the antimicrobial usage patterns in Australia using methods that can be automated to inform antimicrobial use surveillance programs and promote antimicrobial stewardship.

## Introduction

Infections due to microorganisms such as bacteria, fungi, parasites and viruses were a major cause of death until the discovery of antimicrobials [1]. While antimicrobials save countless lives [2], resistance to these drugs has been detected in clinical specimens soon after their introduction to clinical practice [3]. Antimicrobial resistance (AMR) in bacteria has shown a dramatic increase over the last decade and is currently considered an emergent global phenomenon and a major public health problem [4]. Companion animals are able to acquire and exchange multidrug resistant pathogens with humans, and may serve as a reservoir of antimicrobial resistance for in-contact people [5–8]. In addition, AMR is causing poor animal health and welfare outcomes associated with treatment failures in veterinary medicine [9,10]. Knowledge of antimicrobial usage patterns is critical in the implementation and monitoring of antimicrobial stewardship programs. Antimicrobial stewardship (AMS) has been shown to be one of the most effective ways to reduce AMR in a hospital environment [11–14].

VetCompass is a software application that harvests clinical records from veterinary practices into a central repository [15], and is a collaboration between the Royal Veterinary College (RVC) and a consortium of veterinary schools in Australia, focused on improving animal health [16]. This centralized repository of clinical records gives a unique opportunity to examine records that are otherwise held in individual clinics. VetCompass Australia currently has 181 participating practices representing 5.6% of 3,222 of the Australian veterinary clinics [17,18] There have been several studies from VetCompass UK covering antimicrobial usage patterns, disease prevalence, and causes of mortality [19–24]. Similarly, the Small Animal Veterinary Surveillance Network (SAVSNET) is another centralized repository of veterinary records that has also reported on antimicrobial usage patterns [25,26]. The studies from VetCompass UK have used methods which involve the annotation of inventory items of interest by an expert which is a time-consuming and expensive task. Studies from SAVSNET have used methods involving string searching and checking a subset of records. These methods limit the amount of data that can be used, and in doing so, reduce the ability to perform fine-grained *post hoc* analysis of specific cases.

Natural Language Processing (NLP) and automatic text analysis can overcome the challenges of manual labelling of such data, enabling large-scale extraction of key antimicrobial usage information in a structured format, to allow subsequent analysis [27]. NLP is a field of study that sits at the intersection of artificial intelligence and linguistics [28]. The goal of NLP is to automate language analysis, enabling people to communicate more naturally with machines, improving the way humans communicate with each other, or extracting actionable data from text [29]. In this study, NLP was used for text mining, which is the discovery of non-trivial knowledge from unstructured text [30].

The aim of this study was to identify how frequently antimicrobials were prescribed in companion animal practice in Australia, and which antimicrobials were prescribed. This work builds upon our previous work and used previously developed NLP methods [31] to extract antimicrobial usage information in a structured format so we could perform descriptive analysis of the antimicrobial usage patterns of the practices contributing data to VetCompass Australia.

## Materials and methods

De-identified data was sourced from VetCompass Australia (Version 0.3) (2013–2017 inclusive) [18]. Clinical data from 93 practices was required in order to be 95% confident that the estimated rate of antimicrobial usage (AMU) in the 3,222 veterinary clinics of Australia was within 10% of the actual rate of AMU, based on Cochran’s formula for the representativeness of proportions [32]. Antimicrobials were rated according to the antimicrobial importance rating from the Australian Strategic and Technical Advisory Group on Antimicrobial Resistance (ASTAG) which classifies the antimicrobials as low, medium or high importance [33]. Inventory items, which map to all prescriptions and consultation texts, were extracted from the records. Inventory items consist of any item recorded anywhere in the electronic patient record during a visit. Consultation texts include all clinical notes entered in the record in a free text field. Annotations from a subset of data from the state of Victoria were expertly annotated by two veterinarians, for use as the gold standard in assessing the accuracy of the algorithms. A high level of agreement was confirmed between the expert annotators (Fleiss Kappa score of 0.868) [31]. Algorithms were developed and tested on this sample and the most accurate methods for extracting the antimicrobials used in each consult were selected. The created algorithm utilized rule-based logic and a modified version of Levenshtein distance (edit distance, allowing fuzzy string matching) to measure the similarities between individual words [31,34] in order to identify antimicrobial agents in clinical records. The algorithm was determined to have a 96.7% accuracy and an F1 score of 0.85 in extracting the antimicrobial from each consult based on the gold standard annotations [31]. The code developed can be found at: https://www.github.com/havocy28/vetrxmapper. Records were labeled with this algorithm and the distinct items were mapped to their ingredients and ASTAG importance ratings. Inventory items identified as antimicrobial agents were then annotated separately, and reconciled between two veterinarians, as being either topical or systemic medications, along with their World Health Organization (WHO) ratings. Inventory items were imported into a SQL server database and joined to the VetCompass records that had matching item names. As many consultation records were blank with no inventory items associated with them, the consultation table was inner joined to the table with the inventory items associated with them. At least one inventory item and one clinical note combined for a single consultation was required for inclusion in the study. Each inventory item and antimicrobial agent prescribed during a consult is linked to the consultation number and counted as a single consult to the individual patient, regardless of how many items are present in the consult. The postal code of the clinic where each consult took place was mapped to regional descriptions of urban, regional, or suburban using the Australian Bureau of Statistics (ABS) mapping of postal codes [35].

Due to changes in electronic medical record systems, the import of medical records from some practice management software applications did not capture all of the prescription record data, and imported the dispensed medications with descriptions such as “miscellaneous” or “miscellaneous drugs” with no associated prescription labels. Where more than 35% of items were dispensed contained the string ‘misc’ within a given month, these months were excluded from the analysis of that clinic’s records. This included 677 (10%) of the 6,779 months analyzed. Antiprotozoal and antifungal agents were also excluded, as were records where these agents were the only items mapped to the examination note.

Age was reported by subtracting the reported year of birth from the date of the consult, and categorized into yearly increments. Practice usage was reported where there were at least 1,000 consults present. Data was loaded into Microsoft SQL Server 2017 on Linux [36]. All code was written in Python with scikit-learn libraries to perform the machine learning and statistical tests on the algorithms. All descriptive statistics, computations, and visualizations were performed using Tableau 2019.1 [37] with maps of Australia from OpenStreetMap contributors [38]. Significance between values were tested using Pearson’s Chi-squared test.

## Results

A total of 17,797,377 inventory items were identified from the 137 clinics from which sufficient data was available during the study period. These items were mapped to 4,400,519 consultation records. Of the consultation records analyzed, 1,132,986 (26%) records were from 199,358 cats and 3,263,615 (74%) records were from 513,964 dogs. Occasionally, a dog and cat were recorded within the same consultation. There were 595,089 (14%) consultations recorded where antimicrobials were dispensed and 176,243 (4%) consultations where an antimicrobial with high-importance rating was administered or dispensed.

Cat consultations had antimicrobials dispensed in 108 per 1000 consultations, and high-importance rated antimicrobials dispensed in 47 per 1000 consultations. Dogs had higher usage than cats (P < 0.0001) with 145 antimicrobials dispensed per 1000 consultations, but were administered high importance rated antimicrobials in 38 per 1000 consultations, which was less frequently than cats (P < 0.0001).

**Fig 1:**
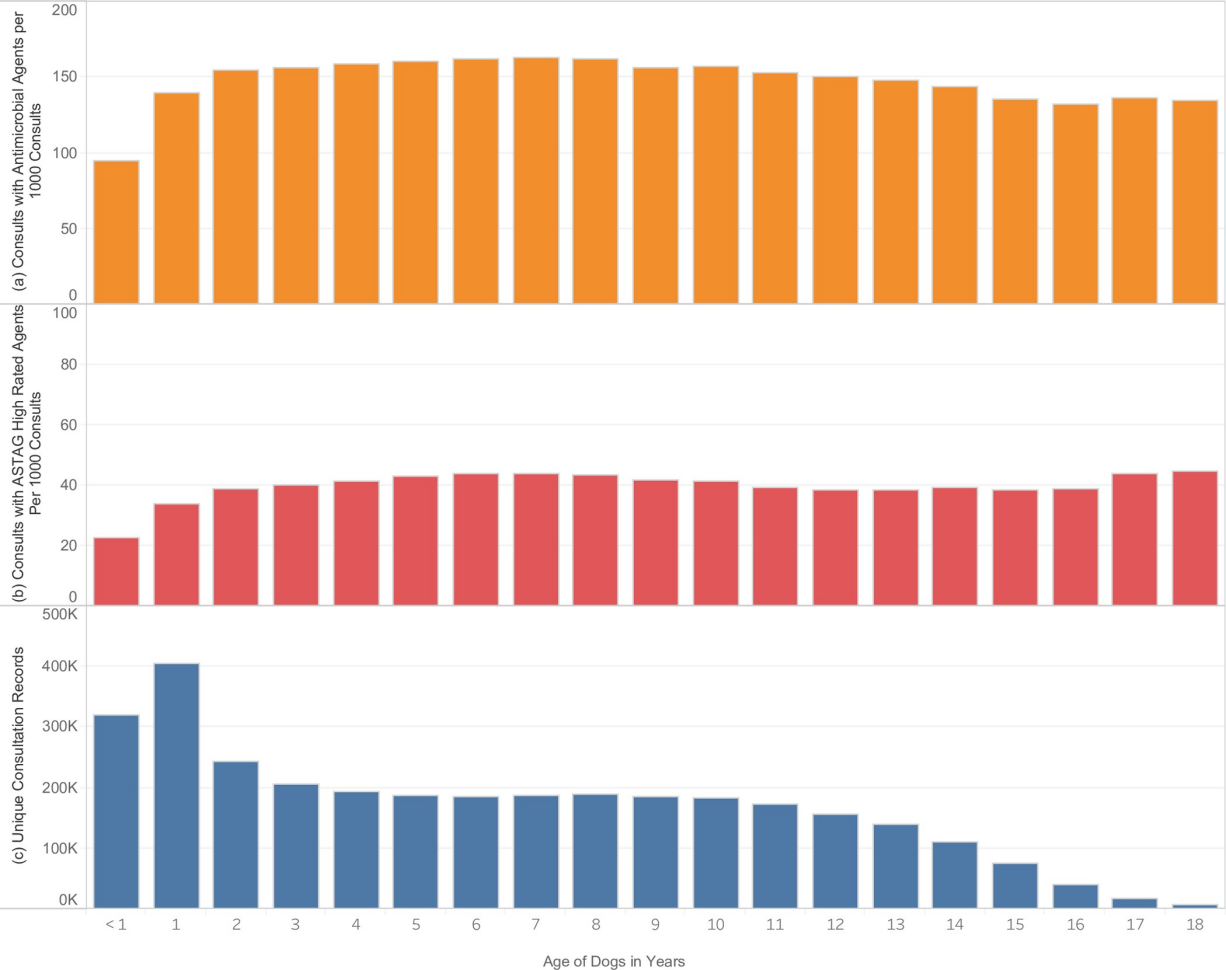
Rate of antimicrobial prescribing in dogs according to age. (A) rate of antimicrobial prescription per 1000 consultations. (B) rate of high-importance antimicrobial prescription per 1000 consultations. (C) number of unique consultations. Years with fewer than 2000 consultations excluded.

**Fig 2:**
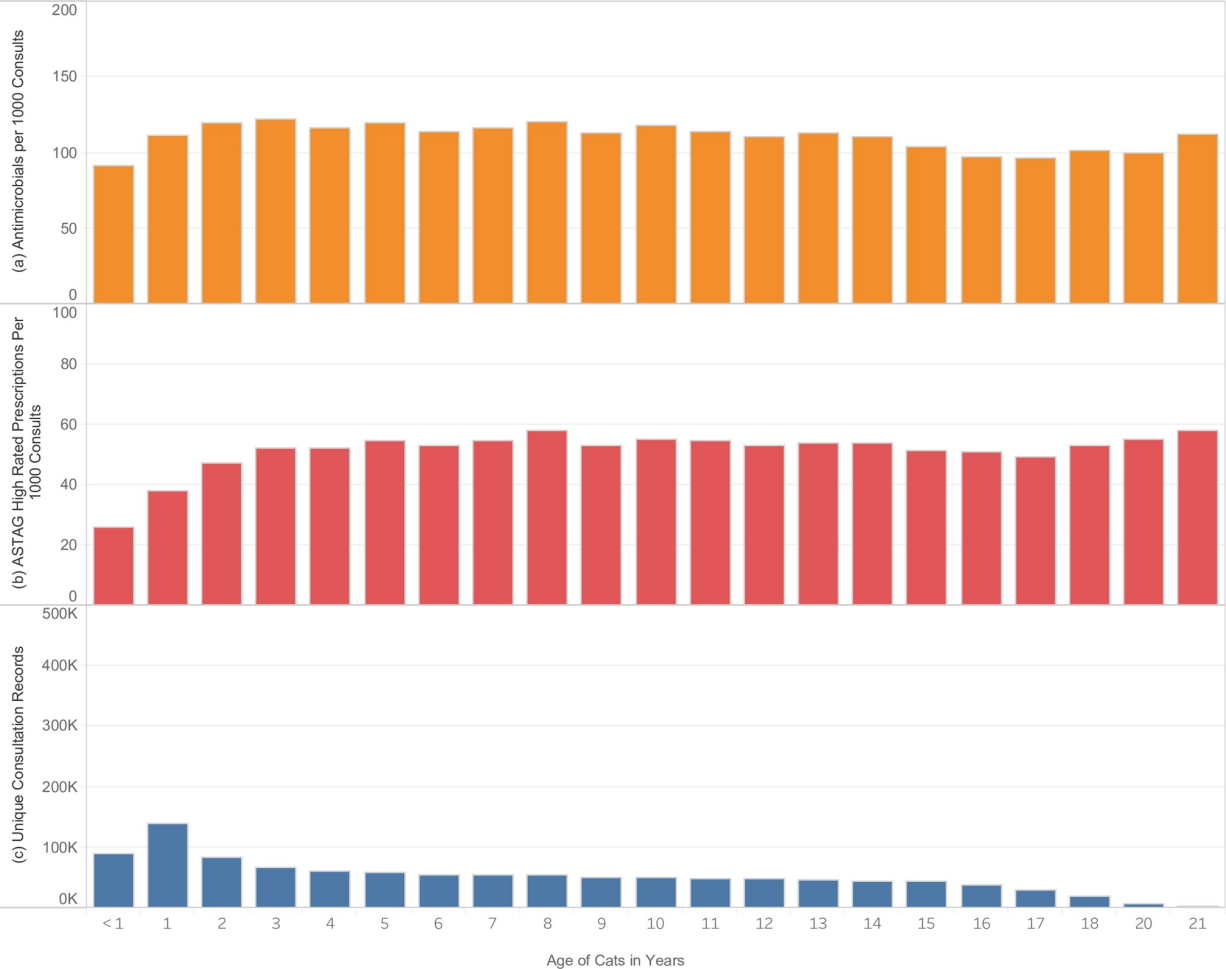
Rate of antimicrobial prescribing in cats according to age. (A) rate of antimicrobial prescription per 1000 consultations. (B) rate of high-importance antimicrobial prescription per 1000 consultations. (C) number of unique consultations. Years with fewer than 2000 consultations excluded.

Dogs less than 1 year in age received significantly fewer antimicrobials overall (95 per 1000 consultations) (Fig 1A), and fewer antimicrobials with high importance rating (23 per 1000 consultations) compared to dogs older than 1 year (P < 0.0001) (Fig 1B). Cats were also prescribed a significantly lower rate of antimicrobials (92 per 1000 consultations) (Fig 2A), and high importance rated antimicrobials (26 per 1000 consultations), during the first year of their lives compared to cats greater than 1 year (P < 0.0001) (Fig 2B).

**Fig 3:**
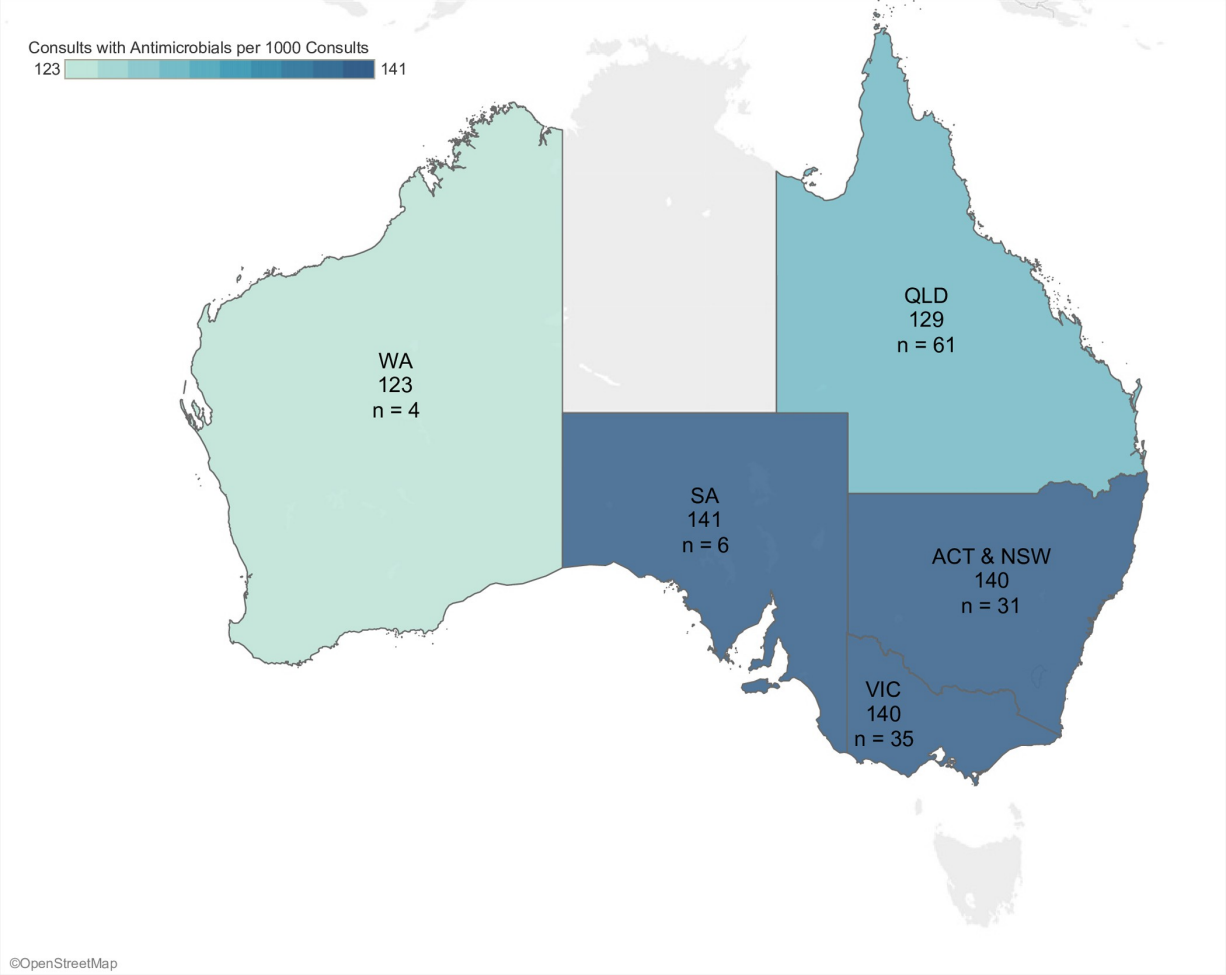
Rate of antimicrobial prescribing by Australian state and territory. n = number of clinics in each region.

**Fig 4:**
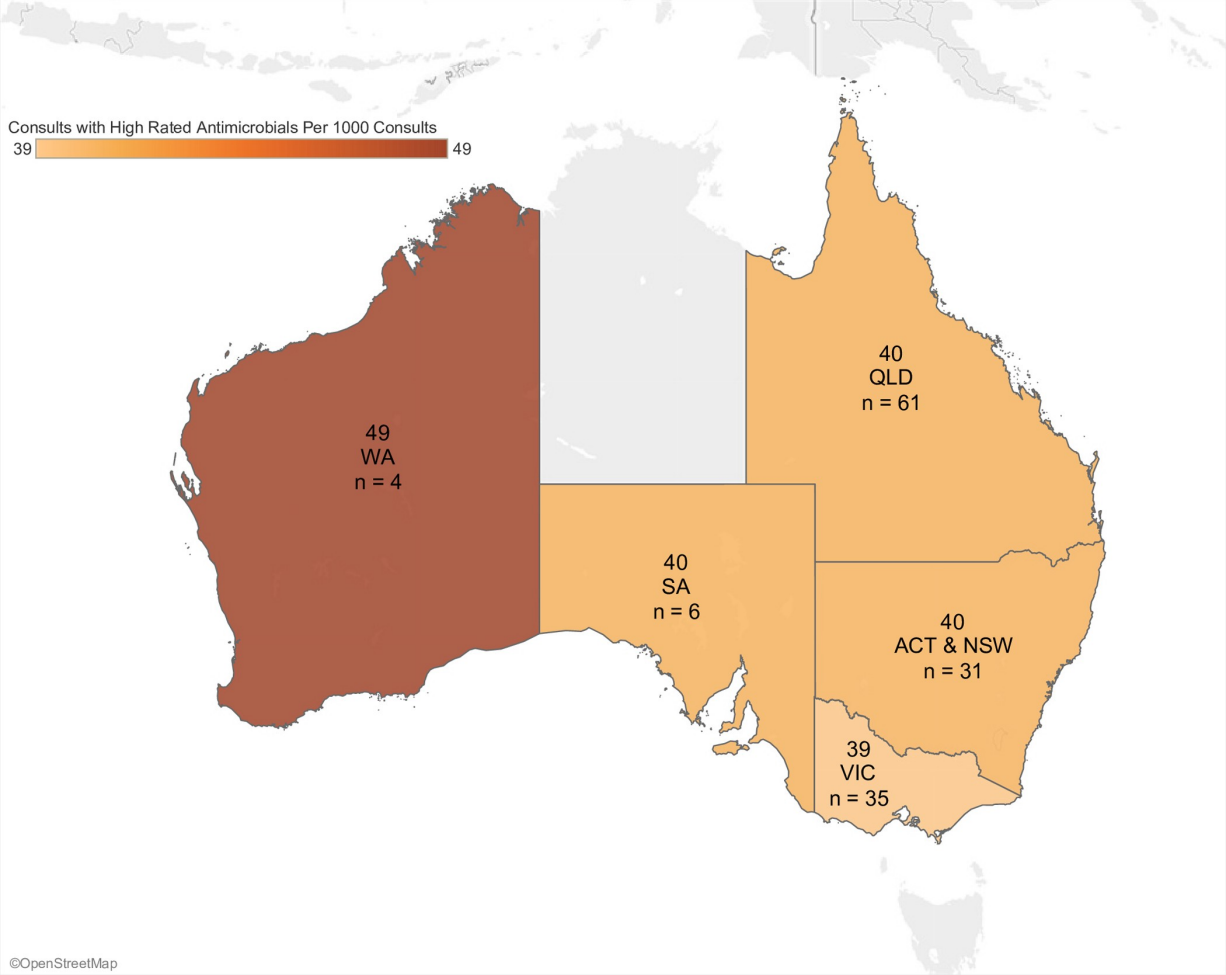
Rate of prescribing of antimicrobials of high-importance rating by Australian state and territory. n = number of clinics in each region.

The range of antimicrobials being administered varied significantly (P < 0.0001) between states from 124 to 141 (median 140) consultations with antimicrobial prescriptions per 1000 consultations (Fig 3). The rate of consultations where antimicrobials with high-importance rating were prescribed varied significantly (P < 0.0001) between states from 39 to 49 (median 40) per 1000 consultations (Fig 4). Antimicrobial use in major cities, inner, and outer regional areas of Australia were different (P < 0.0001) at 136, 138, and 123 consultations with antimicrobial prescriptions per 1000 consultations, respectively. Consultations where high-importance rated antimicrobials were given in inner regional areas, major cities, and outer regional areas of Australia was also different (P < 0.0001), at 41, 35, and 35 per 1000 consultations respectively.

**Fig 5:**
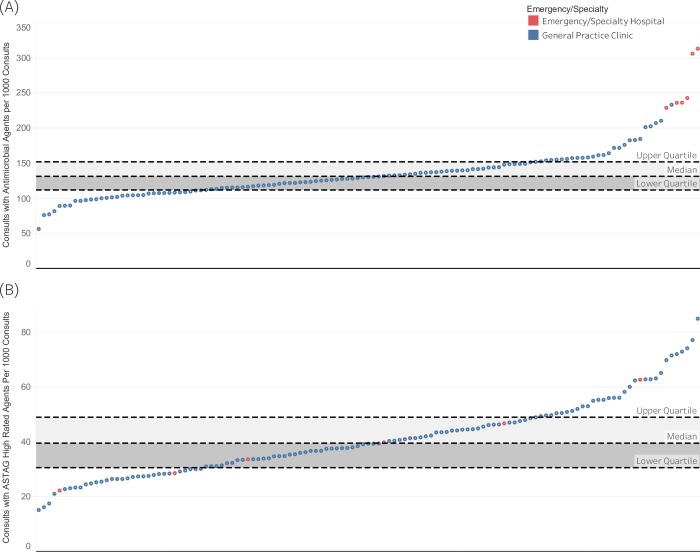
Rate of antimicrobial prescribing in individual clinics. (A) rate of antimicrobial prescription per 1000 consultations. (B) rate of high-importance antimicrobial prescription per 1000 consultations with inter-quartile range is shown in grey. Each dot represents an individual clinic. Clinics with fewer than 1000 consultations excluded.

At the level of individual clinics, 127 clinics matched the selection criteria. The proportion of consultations when at least one antimicrobial was prescribed varied significantly (P < 0.0001), and ranged from 57 times per 1000 consultations to 314 per 1000 consultations (median 131) (Fig 5). Dispensing of antimicrobials with high-importance rating was also different (P < 0.0001) between clinics, ranging from 15 to 85 (median 39) times per 1000 consultations (Fig 5). Emergency and referral centers dispensed antimicrobials 250 times per 1000 consultations and high-importance rated antimicrobials 40 per 1000 consultations. This was higher than general practice clinics (P < 0.0001), which dispensed antimicrobials 132 times per 1000 consultations and high-importance rated antimicrobials 40 times per 1000 consultations.

The most common antimicrobial, and the most common high-importance rated antimicrobial, given to cats was Cefovecin (32% of consultations where antimicrobials were dispensed) (Table 1). The most common topical and high-importance rated topical antimicrobial given to cats was polymyxin B (7.1% of consultations where antimicrobials were dispensed). The most common antimicrobial given to dogs was amoxycillin clavulanate (34% of consultations where antimicrobials were dispensed) (Table 2). The most common high-importance rated antimicrobial dispensed systemically to dogs was enrofloxacin (3.2% of consultations where antimicrobials were dispensed) (Table 2). The most common topical and high rated antimicrobial dispensed to dogs, was polymyxin B (16.9% of consultations where antimicrobials were dispensed). A table with the results of the analysis can be downloaded for further comparisons at: https://havocy28.github.io/am_usage/.

**Table 1:** Frequency of antimicrobials used in cats, antimicrobial agent and administration route (system and topical), importance rating (ASTAG and WHO). Agents with less than 0.01% of the total were omitted. Consults containing both systemic and topical events were counted as one.

Ingredient	ASTAG Rating	WHO Rating	Systemic Antimicrobial Consults (%)	Topical Antimicrobial Consults (%)
Cefovecin	High	Critically Important	39731 (32)	0 (0)
Amoxycillin clavulanate	Medium	Critically Important	37825 (31)	0 (0)
Doxycycline	Low	Highly Important	16143 (13)	0 (0)
Metronidazole	Medium	Important	6372 (5)	0 (0)
Polymyxin B (multi-ingredient)	High	Critically Important	0 (0)	6282 (5.1)
Chloramphenicol	Low	Highly Important	0 (0)	4127 (3.4)
Amoxycillin	Low	Critically Important	3013 (2.5)	0 (0)
Enrofloxacin	High	Critically Important	2910 (2.4)	109 (0.09)
Polymyxin B (single ingredient)	High	Critically Important	0 (0)	2400 (2)
Neomycin	Low	Critically Important	0 (0)	2337 (1.9)
Procaine penicillin	Low	Highly Important	2169 (1.8)	0 (0)
Clindamycin hydrochloride	Medium	Highly Important	1889 (1.5)	0 (0)
Cephalexin	Medium	Highly Important	1840 (1.5)	0 (0)
Cefazolin	Medium	Highly Important	1297 (1.1)	0 (0)
Fusidic acid	High	Highly Important	0 (0)	1183 (1)
Marbofloxacin	High	Critically Important	5790 (0.5)	62 (0.05)
Fusidic acid, framycetin	High	Critically Important	0 (0)	569 (0.5)
Ofloxacin	High	Critically Important	0 (0)	556 (0.5)
Gentamicin	Medium	Critically Important	25 (0.02)	404 (0.3)
Cephalothin	Medium	Highly Important	403 (0.3)	0 (0)
Streptomycin	Low	Critically Important	260 (0.2)	0 (0)
Phthalylsulfathiazole	Low	Highly Important	211 (0.2)	0 (0)
Trimethoprim sulfadiazine	Medium	Highly Important	184 (0.2)	0 (0)
Ampicillin	Low	Critically Important	143 (0.1)	0 (0)
Azithromycin	Low	Critically Important	82 (0.07)	0 (0)
Mupirocin	Medium	Highly Important	0 (0)	65 (0.05)
Ticarcillin clavulanate	High	Critically Important	63 (0.05)	0 (0)
Pradofloxacin	High	Critically Important	55 (0.04)	0 (0)
Neomycin (multi-ingredient) without polymyxin B	Low	Critically Important	0 (0)	50 (0.04)
Framycetin	Low	Critically Important	0 (0)	48 (0.04)
Spiramycin	Low	Critically Important	30 (0.02)	0 (0)
Nitrofurantoin	High	Important	28 (0.02)	0 (0)
Tobramycin	Medium	Critically Important	0 (0)	22 (0.02)
Cephalexin sodium	Medium	Highly Important	21 (0.02)	0 (0)
Cloxacillin	Medium	Highly Important	0 (0)	16 (0.01)
Framycetin, gramicidin	Low	Critically Important	0 (0)	13 (0.01)
Lincomycin	Medium	Highly Important	13 (0.01)	0 (0)
Total			109,719 (89)	18,066 (14)

**Table 2:** Frequency of antimicrobials used in dogs, antimicrobial agent and administration route (system and topical), importance rating (ASTAG and WHO). Agents with less than 0.01% of the total were omitted. Consults containing both systemic and topical events were counted as one.

Ingredient	ASTAG Rating	WHO Rating	Systemic Antimicrobial Consults	Topical Antimicrobial Consults
Amoxycillin clavulanate	Medium	Critically Important	161443 (34)	0 (0)
Cephalexin	Medium	Highly Important	75473 (16)	0 (0)
Metronidazole	Medium	Important	44877 (10)	0 (0)
Polymyxin B	High	Critically Important	0 (0)	42676 (9)
Neomycin	Low	Critically Important	37 (0.01)	40310 (8.5)
Polymyxin B (multi-ingredient)	High	Critically Important	0 (0)	37470 (7.9)
Doxycycline	Low	Highly Important	20963 (4.4)	0 (0)
Enrofloxacin	High	Critically Important	15319 (3.2)	1270 (0.3)
Gentamicin	Medium	Critically Important	390 (0.08)	14946 (3.2)
Cefovecin	High	Critically Important	14678 (3.1)	0 (0)
Chloramphenicol	Low	Highly Important	5 (0)	12235 (2.6)
Cefazolin	Medium	Highly Important	11441 (2.4)	0 (0)
Amoxycillin	Low	Critically Important	9569 (2)	0 (0)
Clindamycin hydrochloride	Medium	Highly Important	5937 (1.3)	0 (0)
Fusidic acid, framycetin	High	Critically Important	0 (0)	5348 (1.1)
Procaine penicillin	Low	Highly Important	4881 (1)	0 (0)
Fusidic acid	High	Highly Important	0 (0)	3941 (0.8)
Cephalothin	Medium	Highly Important	3473 (0.7)	0 (0)
Trimethoprim sulfadiazine	Medium	Highly Important	2760 (0.6)	0 (0)
Ofloxacin	High	Critically Important	0 (0)	2488 (0.5)
Streptomycin	Low	Critically Important	2039 (0.4)	0 (0)
Marbofloxacin	High	Critically Important	506 (0.1)	1189 (0.3)
Phthalylsulfathiazole	Low	Highly Important	802 (0.2)	0 (0)
Ampicillin	Low	Critically Important	428 (0.1)	0 (0)
Neomycin (multi-ingredient [without polymyxin B])	Low	Critically Important	0 (0)	427 (0.09)
Ticarcillin clavulanate	High	Critically Important	302 (0.06)	0 (0)
Spiramycin	Low	Critically Important	290 (0.06)	0 (0
Mupirocin	Medium	Highly Important	0 (0)	211 (0.04)
Cloxacillin	Medium	Highly Important	0 (0)	193 (0.04)
Framycetin	Low	Critically Important	0 (0)	187 (0.04)
Neomycin, nitrofurazone	Low	Critically Important	0 (0)	166 (0.04)
Pradofloxacin	High	Critically Important	142 (0.03)	0 (0)
Tylosin	Low	Critically Important	87 (0.02)	0 (0)
Ceftazidime	High	Critically Important	84 (0.02)	0 (0)
Tobramycin	Medium	Critically Important	1 (0)	77 (0.02)
Lincomycin	Medium	Highly Important	67 (0.01)	0 (0)
Cefotaxime	High	Critically Important	62 (0.01)	0 (0)
Total			343,667 (73)	158,549 (34)

## Discussion

This study is the largest evaluation of antimicrobial prescribing patterns in companion animals to date and gives insight into variation between practices and practice types. Use of Natural Language Processing, and large datasets, allows for the evaluation of antimicrobial prescribing patterns by enabling detailed analysis at the individual consultation level on a per clinic basis. The results demonstrate that while a higher number of antimicrobials are prescribed to dogs, as compared to cats, in cats there is a higher rate of antimicrobials with high-importance rating being dispensed. This is due primarily to the prescribing of cefovecin to cats, a long-acting 3^rd^ generation cephalosporin, consistent with previous research in Australia [39]. However, the previous study of insured pets showed that cats had 47% lower exposure to antimicrobials compared to dogs in any one year, whereas in the current study the difference was only 7.5%. This may be explained by differences in insured and predominately non-insured populations. Higher antimicrobials prescribing in dogs may be due to increased routine preventative health exams performed for cats versus dogs, as reported previously [39]. Further research is needed to investigate these differences. The lower rate of antimicrobial prescribing in younger animals could also be explained by frequent routine appointments for younger pets (vaccination, neutering) that generally do not involve antimicrobial therapy. Further research is required to confirm this.

Difference between states in both the proportion of antimicrobials (12.3% to 14.9%) and high rated antimicrobials (4.0% to 4.9%) may be explained by differences between the types of consultations seen at individual clinics. Variation between clinics and practice types may be due to a higher number of consultations for vaccinations, annual exams, or other routine activities in general practice, which are generally not seen in referral or emergency practices. This variation between practice types is similar in human medicine where emergency practices and some specialties, such as dermatology, have a higher rate of antibiotic prescriptions [40]. Further research is required to investigate these variations.

Considering only consultations where a systemic antimicrobial was dispensed, 47% had amoxicillin clavulanate dispensed, which was very similar to the UK where 45% of patients received amoxicillin clavulanate [41]. This data from VetCompass UK reported antimicrobial events per patient over a period of time compared to individual consultation events reported in our study. This means that patients receiving having multiple consults with antimicrobials during separate consults would only be counted as one event in this UK study, which could account for some of the variation. However, the rate of overall antimicrobial usage for dogs (14%) and cats (11%) varied considerably from a different UK population based on SAVSNET which reported on consultation events, where the figures were 35% for dogs and 49% for cats. However, this study only examined patients presenting with a disease [25] which would reasonably be more likely to require antimicrobials. Polymyxin B was given at a relatively high rate in both dogs (16.9%) and cats (7.1%) topically. Veterinary usage of polymyxin B has previously come under scrutiny for its oral administration in food animals [42,43]. Further research is required to better understand the significance, if any, of polymyxin B usage in companion animals and its contribution to antimicrobial resistance more broadly.

Of the antimicrobials with high-importance rating, cefovecin was the most frequently administered (16% of all antimicrobials). This was primarily due to the high usage in cats (32%) as it was used ten times less frequently in dogs (3.1%). Only 3 other agents of high-importance rating administered systemically had greater than 0.1% rate of administration: the fluoroquinolones enrofloxacin, marbofloxacin, and ofloxacin. Of these fluoroquinolones, enrofloxacin was used the most frequently, but still only represented 3.3% of all the antimicrobials being dispensed (3.5% for dogs). Importantly there were no glycopeptides, such as vancomycin, or carbapenems, such as imipenem, found in the dataset, which are generally thought of as the last line of antibiotics in human medicine [44].

The methods used in this study enhance the ability to use large-scale data and overcome some of the limitations previously encountered due to a lack of standards in fields, where data was entered into the electronic records [41]. NLP methods for extraction of prescription information have been well documented in various studies in human medicine [45], however relevant clinical practice data is difficult to access from the medical sector at a large scale due to privacy considerations. Central data repositories such as VetCompass and SAVSNET help overcome this issue in veterinary medicine, allowing for an approach using NLP that benefits both human and veterinary medicine by demonstrating their applicability to address important questions about real-world practice patterns. The implementation of these algorithms to extract antimicrobials out of prescriptions is the largest study at the time of this paper. By using algorithms to match antimicrobials, and verifying the accuracy of these algorithms through expert annotation of the original records, we have been able to perform data analysis on the antimicrobial usage patterns on a very large number of clinical records.

Data analysis performed in this study was limited to version 0.3 of VetCompass-Australia. There are also some records that are linked to the wrong patients, caused by duplicate IDs within the database; this was estimated to effect < 0.01% of the records. Our data was obtained at the consult level, meaning that describing the number of antimicrobials received at the individual patient level was not possible, as some patients may attend other veterinary clinics not recorded in VetCompass. The reason that a patient was prescribed antimicrobials was not analyzed for this study as this required analysis of the free text of the medical record, which was not labeled, and was therefore outside the scope of this study. Further methods are being developed currently to undertake analysis of the clinical records to determine the reason for the consults and appropriateness of antimicrobial use. Additionally, the size of the sample in this study results in very small differences becoming “statistically significant”. This is a common issue with big data [46–48]. In this scenario, the clinical significance of results becomes much more important and should be considered in the interpretation of results [46].

## Conclusion

Utilizing Natural Language Processing and VetCompass Australia, we have created a detailed analysis of antimicrobial usage on a per clinic basis. Overall, approximately 14% of consultations had antimicrobials dispensed, and in 3.9% of consultations antimicrobial of high-importance rating were administered or dispensed. The most common antimicrobial dispensed to dogs was amoxycillin clavulanate (34%), while cefovecin (32%) was the antimicrobial agent most frequently administered to cats. The most common antimicrobial of high-importance rating administered to cats and dogs was cefovecin and enrofloxacin, respectively. These results provide a description of the usage over a 5-year period that can be used to inform changes in practice prospectively, and can support the continued surveillance of antimicrobial usage in companion animal veterinary practices in Australia.
